# Sense and Sensibilities of Organ Perfusion as a Kidney and Liver Viability Assessment Platform

**DOI:** 10.3389/ti.2022.10312

**Published:** 2022-03-14

**Authors:** Laurence Verstraeten, Ina Jochmans

**Affiliations:** ^1^ Lab of Abdominal Transplantation, Transplantation Research Group, Department of Microbiology, Immunology and Transplantation, KU Leuven, Leuven, Belgium; ^2^ Department of Abdominal Transplantation, University Hospitals Leuven, Leuven, Belgium

**Keywords:** kidney transplantation, liver transplantation, hypothermic perfusion, machine perfusion, organ preservation, normothermic perfusion, organ viability assessment

## Abstract

Predicting organ viability before transplantation remains one of the most challenging and ambitious objectives in transplant surgery. Waitlist mortality is high while transplantable organs are discarded. Currently, around 20% of deceased donor kidneys and livers are discarded because of “poor organ quality”, Decisions to discard are still mainly a subjective judgement since there are only limited reliable tools predictive of outcome available. Organ perfusion technology has been posed as a platform for pre-transplant organ viability assessment. Markers of graft injury and function as well as perfusion parameters have been investigated as possible viability markers during *ex-situ* hypothermic and normothermic perfusion. We provide an overview of the available evidence for the use of kidney and liver perfusion as a tool to predict posttransplant outcomes. Although evidence shows post-transplant outcomes can be predicted by both injury markers and perfusion parameters during hypothermic kidney perfusion, the predictive accuracy is too low to warrant clinical decision making based upon these parameters alone. In liver, further evidence on the usefulness of hypothermic perfusion as a predictive tool is needed. Normothermic perfusion, during which the organ remains fully metabolically active, seems a more promising platform for true viability assessment. Although we do not yet fully understand “on-pump” organ behaviour at normothermia, initial data in kidney and liver are promising. Besides the need for well-designed (registry) studies to advance the field, the catch-22 of selection bias in clinical studies needs addressing.

## Introduction

One of the underlying causes of the perpetuating organ shortage is the discarding of transplantable organs based on “poor organ quality”. Currently, up to 20% of kidneys and 10% of livers that are recovered in the United states are not transplanted (1). Eurotransplant data show similar figures for kidney with considerably lower utilization rates for livers donated after circulatory death (DCD) compared to those donated after brain death (DBD) (2). A major contributor to organ discard is the fact that organ quality and viability remain difficult to predict accurately (1). With the increasing use of DCD kidneys and livers, the need for reliable pre-transplant viability assessment has become even more important. Indeed, DCD kidneys suffer from higher rates of delayed graft function (DGF) and primary non function (PNF) leading to a significant morbidity and mortality risk for the recipient (3, 4). DGF is associated with an increased risk of acute rejection, longer in hospital stay, higher cost and lower graft survival (5, 6). Higher-risk liver grafts, especially those from DCD donors, suffer higher incidences of PNF and intrahepatic cholangiopathy ultimately leading to higher graft failure rates compared to DBD livers (7, 8).

**FIGURE 1:**
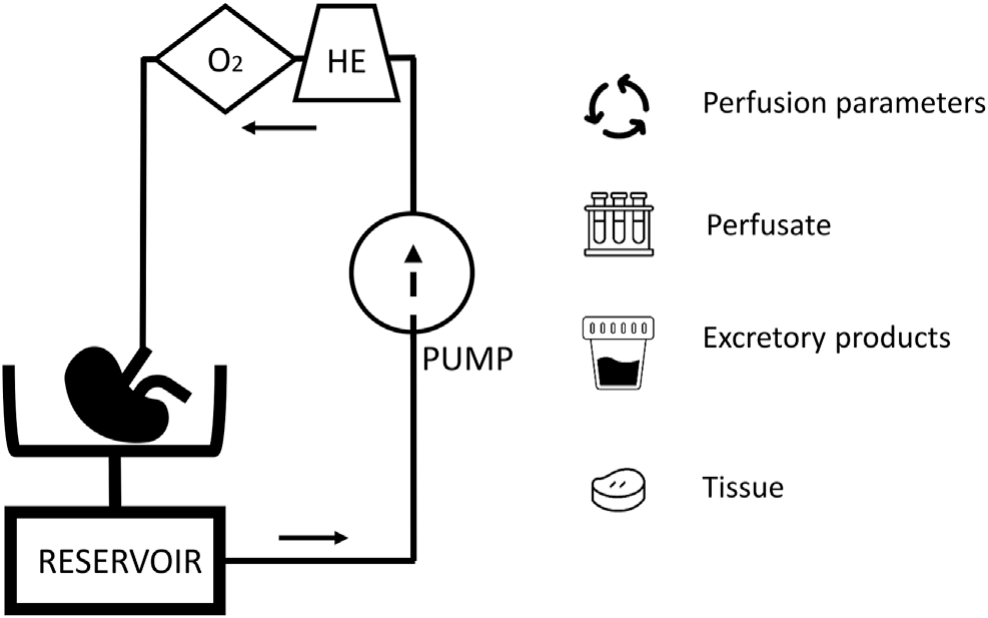
A schematic overview of a kidney perfusion circuit: The presence of a heat exchanger (HE) and gas exchanger (O_2_) depends on the perfusion mode. During perfusion, perfusion parameters, perfusate, excretory products (e.g., urine during normothermic perfusion), and tissue are available for viability assessment.

While with static cold storage, only limited options to assess organ function and viability are available, organ perfusion preservation has been posed as a platform for organ viability assessment (9). During organ perfusion, a perfusion solution is circulated through the vasculature, driven by a pump. The perfusion solution can be cooled or heated and, often with the help of a gas-exchanger, oxygenated. During hypothermic perfusion an acellular perfusion solution is used, in normothermic conditions an oxygen carrier is needed and this are often red blood cells. In this dynamic environment, the organ can be assessed real-time by evaluating perfusion parameters and injury markers (Figure 1). When the organ is metabolically active, markers of organ function can also be studied. As (patho)physiology involves a complex interplay of different cells, it is likely that true prediction of organ viability will need the assessment of more than a single parameter.

This review provides an overview of the available clinical evidence on the use of organ perfusion as a platform to predict kidney and liver viability before transplantation.

## Kidney

Hypothermic kidney perfusion became a clinical reality after much preclinical work in the 1960s by pioneers like F.O. Belzer (10–12). Due to refinement of preservation solutions good results with the cheaper and simpler static cold storage were obtained and kidney perfusion disappeared to the background. Nevertheless, hypothermic kidney perfusion has been reintroduced in clinical settings after it was shown to reduce the risk of DGF compared to static cold storage (13). Normothermic perfusion is being investigated in research settings with a first randomised trial underway (14).

### Pathophysiology of the Ischemic Injury

To assess kidney viability, understanding the pathophysiology of ischemia reperfusion injury is crucial. Every transplantation procedure is associated with ischemia reperfusion injury that impacts post-operative tissue injury and graft function. The biological pathways behind ischemia reperfusion injury describe functional and structural changes in the organ based on changes in cell metabolism (especially in the mitochondria). Various molecular mechanisms are active in ischemia reperfusion injury. There is the critical role of the anaerobic metabolism during ischemia, resulting in intracellular acidosis, ATP depletion, and failure of ion-exchange channels, setting the stage for reperfusion injury (15). Post-reperfusion, innate and adaptive immune responses are activated by reactive oxygen species and damage associated molecular patterns, resulting in a sterile inflammation (16–19). Ischemia reperfusion injury causes structural and functional damage to renal tubules by inducing tubular cell death which manifests as a clinical spectrum of acute kidney injury ranging from transient acute kidney injury to primary non-function (PNF). When the transient acute kidney injury is severe enough, and the patient needs dialysis in the first week after transplantation, delayed graft function (DGF) occurs. An association between DGF and acute rejection has been reported (20) and this might affect long-term graft function as persistent inflammation in scarred areas after T-cell mediated rejection has been associated with chronic scarring and fibrosis due to maladaptive injury responses (21).

This injury process leaves marks, e.g., representing epithelial cell disruption and tubular injury that might be detected as biomarkers in the perfusate (22, 23).

### Hypothermic Kidney Perfusion

In hypothermic conditions, options to assess kidney function are limited. Indeed, the metabolic rate at 4°C is limited to 10% of that at physiological temperature with a 40% lower rate of chemical reactions (24). Furthermore, in the majority of cases there is no active oxygenation during hypothermic kidney perfusion in which case aerobic metabolism is not supported (25). Focus has therefore been on identifying associations between markers of injury and post-transplant outcome.

#### Perfusate Injury Markers

Injured tubular cells release proteins into the perfusate during hypothermic perfusion where they can be detected. Today, there is good quality evidence that perfusate injury markers should not be used to assess viability of kidneys during standard hypothermic organ perfusion. In a systematic review, Guzzi et al. summarized the findings of 29 clinical studies assessing the association between PNF, DGF, and long-term graft survival and perfusate injury markers measured during hypothermic perfusion of DCD and DBD kidneys (26). Only four studies were identified as good quality prospective studies (27–30).

Glutathione S-Transferase (GST) concentrations during hypothermic perfusion have been well-studied with an independent association with DGF, however, the predictive accuracy of GST for DGF is moderate at best and no correlation with long-term outcome has been found (27–29). Similar to GST, perfusate lactate dehydrogenase independently associates with DGF and PNF but predictive accuracy is low (27, 31). While heart-type fatty acid binding protein (H-FABP) showed to be an accurate biomarker of kidney injury after transplantation in preclinical studies (32), clinical studies showed only moderate predictive power of perfusate H-FABP for DGF (27, 31). Neutrophil gelatinase-associated lipocalin (NGAL), released by renal tubular cells in response to ischemic injury, is a recognized biomarker of acute kidney injury (26, 30, 31, 33), but no reliable association of NGAL release during hypothermic perfusion with post-transplant outcomes has been found (31). Some studies assessing perfusate lipid peroxidation and perfusate interleukin-18 (a pro-inflammatory cytokine) show little promise as viability markers (28, 31). Associations between other biological parameters, like lactate, N-acetyl-D-glucosamine, Kidney injury molecule 1, and others were either not significant, not accurate, or described in single studies. Growing interest in microRNA’s in multiple disease processes draws our attention for their use in viability assessment during hypothermic perfusion (34).

Whether predictive accuracy of perfusate injury markers is improved when the perfusate is actively oxygenated, is not known and subject of ongoing research (www.cope-eu.org). This is an important question as hypothermic oxygenated perfusion is already finding its way into clinical practice (e.g., the Netherlands) after it was recently shown that older DCD kidneys benefit from active oxygenation in the cold (25).

#### Perfusion Parameters

Since the early days of hypothermic kidney perfusion, it has been hypothesised that kidney viability is associated with perfusion parameters. Indeed, at a stable perfusion pressure, a lower renal flow indicates a higher intrarenal resistance and reflects increased vascular injury or interstitial oedema. A correlation between perfusion parameters and DGF and PNF has been shown in retrospective studies that suffered from selection bias as kidneys were discarded based upon perfusion parameters(35–38). A large randomized controlled prospective trial, without selection bias, has shown that renal resistance at the end of hypothermic perfusion is an independent risk factor for DGF and 1-year graft survival but the predictive accuracy is low (39). These findings have been confirmed by Parikh et al. in a large prospective cohort (30).

While perfusion parameters, such as renal resistance on the pump, provide additional information on quality of the graft, they should not be used as clinical decision making tools. When the perfusate is actively oxygenated, endothelial cell integrity might be improved. This might change perfusion parameters and their predictive power which is the subject of ongoing research (www.cope-eu.org). In addition, in relating Ohm’s Law to fluid flow (Eq. 1), it is important to remember that exact flow or resistance values will depend not only on the kidney but also on the perfusion device (pressure or flow driven) and the settings (e.g., pump pressure chosen) that are used. Perfusion parameter read-outs, and therefore any defined thresholds, are not necessarily transferable from one perfusion device to the other.

ΔP/F=R (1)

where ΔP is the driving pressure of perfusion pressure as set by the pump (in mmHg) in case of a pressure-controlled system, F is renal artery flow (ml/min), and R is the renal resistance (mmHg/mL/min).

### Normothermic Kidney Perfusion

The advantages of normothermic perfusion with regard to viability assessment relate to the use of a perfusate based on oxygenated red blood cells or oxygen carriers at physiological temperatures, meaning the graft can be fully metabolically active. In addition to assessing injury markers and perfusion parameters, normothermic perfusion would therefore allow to evaluate kidney function. Indeed, e.g., creatinine can be added to the perfusate and in this way a creatinine clearance from the perfusate over time can be calculated. In contrast to hypothermic perfusion, normothermic perfusion requires considerable technical expertise with the potential of dramatic consequences in case of technical failure as the graft would be exposed to warm ischemia.

Normothermic perfusion as mostly been developed to be used as a “resuscitation tool.” This means a short period (1–2 h) of normothermic perfusion immediately before transplantation following static cold storage (40). Results of a first randomised controlled phase II trial assessing the effectiveness of normothermic perfusion as a resuscitation tool compared to static cold storage in controlled DCD kidneys are awaited (41). Meanwhile, experimental data show the feasibility, and possible benefit, of prolonged normothermic perfusion preservation starting immediately after kidney procurement (42, 43).

Initial evidence that normothermic perfusion could be used as a platform to assess viability pre-transplantation was provided by Hosgood et al. when a discarded kidney was transplanted after evaluation during a short period of normothermic perfusion (44). In a further series of kidneys, that were considered unsuitable for transplantation, a kidney quality score during normothermic perfusion was derived. This score is based on the macroscopic aspect of kidneys during perfusion, the arterial flow, and volume of urine production. Kidneys with a score ≥3 out of 5 were considered transplantable (Table 1) (44–46). The clinical studies leading up to development of the score suffered from selection bias because not all kidneys were transplanted. The score remains to be validated in large series. In that light, it is important to realise that the majority of the evidence on the use of normothermic perfusion as a viability assessment platform has been obtained from kidneys that were perfused on a custom made circuit. Therefore, the threshold flow values as proposed by Hosgood et al. depend on the perfusion pressure (Eq. 1) (47) and are not directly transferable to settings using different perfusion pressures. Also, although the physical properties of the filter remain the same when a healthy kidney is perfused *ex situ*, the perfusate composition and perfusion pressures (pump pressures) will change oncotic and hydrostatic pressures and therefore influence filtration and ultimately “urine” production during kidney perfusion (48). Adding tubular injury markers to the kidney quality assessment score might improve its accuracy and this has been explored (49).

**TABLE 1:** Kidney quality assessment score as defined by Hosgood et al.

Kidney quality assessment score parameter	Point
Macroscopic assessment	
Grade I: Excellent perfusion (global pink appearance)	0
Grade II: Moderate perfusion (patchy appearance)	1
Grade III: Poor perfusion (global mottled and purple/black appearance)	2
Renal Blood flow	
Threshold ≥50 ml/min/100 g	0
Threshold <50 ml/min/100 g	1
Total urine output	
Threshold ≥50 ml/min/100 g	0
Threshold <50 ml/min/100 g	1

Scores range from 1 to 5, 1 indicating the least injury to 5 the most severe. Reproduced from (95) with permission under Creative Commons Attribution 4.0 International License (http://creativecommons.org/licenses/by/4.0).

Importantly, Schutter et al. recently showed that early functional assessment may not reflect actual physiology. In a pig model of normothermic perfusion kidneys were mainly centrally perfused in the first 2 h of perfusion, while it took time for the outer cortex to reach its physiological dominant perfusion state (50). Before that, the functionally important renal cortex appeared severely underperfused, meaning longer perfusion times might be needed for reliable viability assessment. This point was also raised by Hosgood et al. who recently published a report on a pair of kidneys that had passed the quality assessment test but still developed PNF (51).

## Liver

In contrast to kidney perfusion, liver perfusion has not yet reached the stage of wide-spread clinical implementation. Building on the pioneering work of Starzl and others (52–54), both hypothermic and normothermic liver perfusion are now the topic of several clinical studies investigating the value of perfusion as a preservation method but also as a platform for organ viability assessment. The need for optimized preservation and reliable viability assessment is high as an increasing number of DCD livers, at higher risk of PNF and post-transplant cholangiopathy, are offered for transplantation (7, 8). Like in the kidney, ischemia reperfusion injury in the liver causes cellular injury. Hepatocellular injury leads to a spectrum of clinical presentation, marked by increased transaminases. When severe, early allograft dysfunction occurs which is associated with increased mortality and graft loss (55–57). When irreversible, in the case of PNF, recipient mortality is high (58). While the liver regenerates, it remains difficult to assess what level of injury a liver can tolerate while still providing life sustaining function to the recipient. Furthermore, cholangiocyte injury and injury to the peribiliary plexus can lead to post-transplant cholangiopathy, a vexing complication leading to increased morbidity and reduced graft survival (59, 60). Liver perfusion offers a window of opportunity to gather additional information on both the level of injury sustained and the remaining liver function.

### Hypothermic Liver Perfusion

Like in kidney, options to assess liver function during hypothermic perfusion are likely limited because metabolic rate is severely reduced. However, in contrast to kidney, hypothermic liver perfusion is nearly always actively oxygenated and mitochondrial respiration continues (61). A short period of hypothermic oxygenated perfusion of the liver has been described to have immunomodulatory effects, preserve the endothelial cell glycocalyx and the peri-biliary vascular plexus and glands, and improve post-transplant outcomes (61–65). Recent studies have shown less post-transplant hepatocyte injury and reduced cholangiopathy rates with hypothermic oxygenated perfusion (65, 66).

#### Perfusate Injury Markers

In the first clinical series of hypothermic liver perfusion, Guerrera et al. already described a correlation between perfusate and post-transplant serum transaminases (63, 67). These findings were confirmed by Patrono et al. but none of the injury markers were independently associated with outcomes (68). The detection of mitochondrial flavin mononucleotide (FMN), an integral part of mitochondrial complex I, in the perfusate might be a surrogate marker for impaired cellular energy production.

There is evidence that the release of FMN occurs independently of the other hepatocellular enzymes (69). A strong correlation of FMN with post-transplant peak transaminases and coagulation factors was found in addition to correlation of FMN with hospital stay, post-transplant complications, and graft failure within 3 months (69). FMN was also predictive of early allograft dysfunction (69). The correlation of FMN with early allograft dysfunction was also described by Patrono et al. though not found to be significant (62). Currently there is too little evidence to conclude whether injury markers measured during hypothermic oxygenated liver perfusion are helpful in predicting viability. With the completion of the first large trials, further evidence on the proper value of these markers is likely to become available in the near future (NCT01317342) (65).

### Perfusion Parameters

Very little is known about the relationship between hepatic artery or portal vein flow and resistance during hypothermic oxygenated liver perfusion. Like in the kidney, an increase in flow over time and a decrease of hepatic artery resistance are observed (65, 70). Patrono et al. observed a slower decrease in hepatic artery resistance in livers that developed early allograft dysfunction but larger series need to be analysed to understand the value of perfusion parameters as predictor of post-transplant viability (70).

### Normothermic Liver Perfusion

In contrast to normothermic kidney perfusion, normothermic liver perfusion is more widely studied. In a randomised study, normothermic liver perfusion has been shown to reduce post-transplant graft injury, measured by hepatocellular enzyme release, compared to cold storage (71). Despite these findings, no differences were seen in graft or patient survival, hospital stay and bile duct complications. Remarkably, a 50% lower rate of organ discard was noticed in the perfusion arm, confirming the need for pre-transplant viability assessment to increase the number of liver transplants. It must be noted that this trial was not designed to address organ utilization and selection bias because of the non-blinded nature might have been present. Trials with organ utilization as primary outcome should randomise as early in the process as possible, ideally at the time of the organ offer or even at the time of listing the patient for transplant (72). A short period of normothermic liver perfusion to test viability has been explored by a number of groups (73–82). Encouraging results have led to the implementation of normothermic liver perfusion as a viability assessment tool in expert centres although there is considerable variability in both indications and assessment criteria (83). Because the liver is metabolically active, liver function might be assessed during normothermic perfusion. In this light it is important to remember that both hepatocytes and cholangiocytes need to be functioning for sustained graft function and survival.

#### Markers of Hepatocyte Injury and Function

**FIGURE 2:**
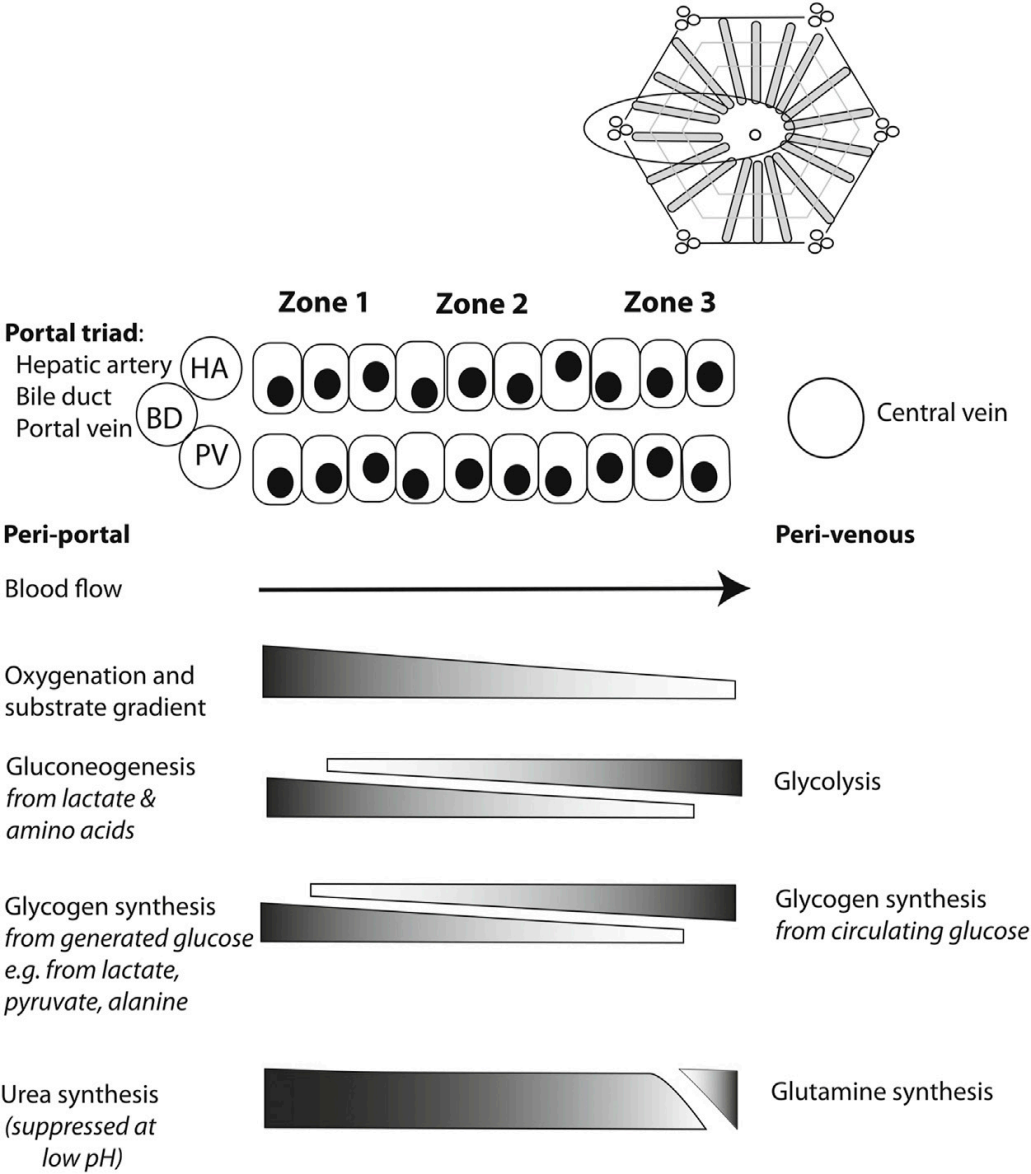
Schematic overview illustrating metabolic liver zonation with reference to glucose and ammonia metabolism. Blood entering the liver lobule *in vivo* through hepatic artery (HA) and portal vein (PV) branches is rich in hormones, nutrients and oxygen. Periportal (zone 1) metabolic processes will include those requiring such conditions, while perivenous (zone 3) hepatocytes may preferentially include those metabolic processes that are less dependent on high levels of oxygen, for example, or those requiring products made in the periportal hepatocytes, such as urea. Reproduced from (84) with permission under Creative Commons Attribution 4.0 International License (http://creativecommons.org/licenses/by/4.0).

In assessing the hepatocyte compartment, the zonation of the hepatocytes helps when interpreting the meaning of several perfusate markers (84). As oxygen concentrations are the highest in the periportal zone, zone 1 hepatocytes are differentiated to carry out processes that require high oxygen concentrations (Figure 2). Near the central vein, zone 3 hepatocytes are adapted to the low oxygen concentrations that are present.

### Perfusate Transaminases

**FIGURE 3:**
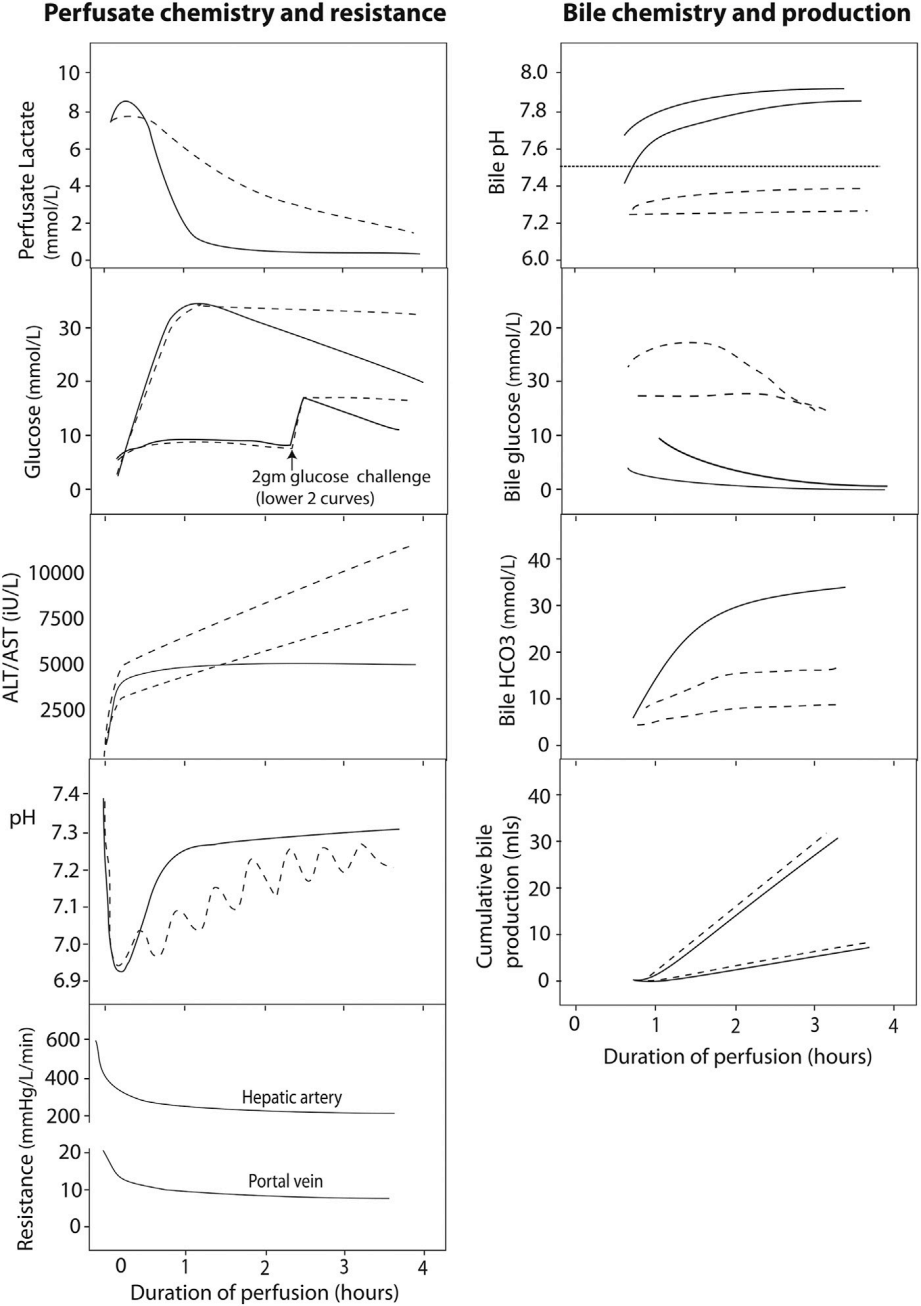
Typical normothermic perfusate profiles of liver perfusion. The figure shows schematic graphs with typical biochemical and resistance profiles during normothermic liver perfusion with an interpretation regarding viability given according to current state of knowledge. Profiles of viable hepatocellular compartment livers are denoted by solid black lines, while dashed lines denote grafts where viability might be in doubt, due to a slow lactate clearance, persistently raised perfusate glucose, rising perfusate transaminase concentration or requirement for continued bicarbonate support to maintain pH. The graphs also show the different biochemical profiles of bile depending on the viability of the ducts, where viable cholangiocytes producing bile with an alkaline pH, low glucose (especially relative to the high perfusate glucose) and increasing bicarbonate levels. To date, there is no clinical evidence in support of bile production or hepatic resistance thresholds for viability. Reproduced from (84) with permission under Creative Commons Attribution 4.0 International License (http://creativecommons.org/licenses/by/4.0).

Perfusate transaminases (as opposed to postoperative systemic levels of transaminase) have been used to determine the viability of a particular graft for implantation. In viable livers, perfusate transaminases seem to plateau over time. Most livers will reach this plateau by 2 h (76, 77, 82) therefore continued transaminase increase is suggestive of ongoing injury during perfusion (Figure 3). It must be noted that transaminase levels may be influenced by the age of the donor, steatosis, ischemia time, among other factors (72). Perfusate transaminases should be normalized for liver weight and perfusate volume to allow comparability with other perfusion systems and different livers (72). Because aspartate aminotransferase may also rise from haemolysis on the circuit, alanine aminotransferase might be more representative of the degree of hepatocellular damage (76, 77, 85).

Perfusate transaminases seem to be correlated with post-transplant systemic levels of transaminases (77) though the usefulness of this correlation in helping predict outcome is unclear. Indeed, postoperative levels of transaminases are influenced by the perfusion itself and the large volume of perfusate (wash-out) (72). Additionally, bilirubin and INR seem to have a stronger predictive capacity for patient and graft survival compared to AST, indicating that hepatocyte injury with little involvement of the biliary tree has a more benign course (86). The usefulness of the current definition of early allograft dysfunction (using peak transaminases in the first week, total bilirubin and INR levels) (55) in case of livers transplanted after perfusion is unclear and the definition might need revisiting (86, 87).

#### Perfusate Lactate

A slow clearance of lactate is associated with severe parenchymal injury where viability may be in doubt (71, 73, 77, 84, 85). Indeed, lactate metabolism occurs mainly in the periportal hepatocytes (zone 1), so a viable rim of zone 1 hepatocytes can metabolise the lactate in the relatively small volume of perfusate, even in the presence of severe parenchymal damage in zone 2 and 3 (Figure 3). Therefore, lactate is not recommended as a single viability marker.

#### Perfusate Glucose

Glycogenolysis is an ATP-independent process that continues during cold storage, evidenced by increasing perfusate glucose levels early during normothermic perfusion (Figure 3). A normal level of glucose during normothermic perfusion may reflect minimal ischemia, but may point out glycogen exhaustion or extensive liver injury (77). Over time, a viable liver will re-incorporate this glucose into glycogen during perfusion (Figure 3) (77).

#### Acid-Base Homeostasis During Perfusion

Regulation of the hepatic acid-base balance depends, among others, upon the differential metabolism of glutamine along the lobule (88). Healthy livers tend to have a better pH regulation and stabilisation (Figure 3). Analysing pH and the need for external regulation by bicarbonate replacement could help assessment viability of the hepatocyte compartment (76, 77).

#### Coagulation Factors During Perfusion

In a preclinical study, severely injured livers have low perfusate levels of anticoagulant and coagulation factors compared to those that are minimally injured livers (89). Little information on the value of perfusate (anti)coagulation factors in human settings is available. Such proteins are detectable but no correlation between (anti)coagulation factors and severity of post-transplant injury has been shown (89, 90). Whether low factor concentrations are predictive of outcome remains to be investigated (89).

#### Bile Production During Perfusion

Bile production is an important function of the hepatocyte and the volume of bile produced during normothermic perfusion has been associated with hepatocyte injury (91). However, the absence of bile production during perfusion is not necessarily a feature of a non-viable graft (71, 92).

#### Markers of Cholangiocyte Injury and Function

The importance of assessing cholangiocyte viability was recently demonstrated by Mergental et al. who selected livers, thought unsuitable for transplantation on static cold storage, based on hepatocyte viability criteria. Of 31 initially discarded livers, 22 (71%) met hepatocyte viability criteria were successfully transplanted with no PNF cases. However, three out of ten (30%) DCD livers developed biliary complications requiring retransplantation (80). Indeed, while the hepatocyte is responsible for producing bile, the healthy cholangiocyte ensures an alkaline composition of bile with low glucose levels (Figure 3) (92, 93). Watson et al. and Matton et al. provide suggested cut off values for bile pH, glucose, and bicarbonate concentrations that need validation in large series (77, 78, 85, 94). As for kidney, clinical studies identifying these cut-off values suffer from selection bias as not all livers were transplanted, though pathological assessment of the intra-hepatic bile ducts of some of the non-transplanted livers were correlated with bile biochemistry (77).

#### Perfusion Parameters

Hepatic artery and portal vein resistance decrease quickly during perfusion to reach a steady state (Figure 3). Little is known about the meaning of these findings though Watson et al. observed no correlation of these parameters with outcome or biochemical markers of hepatocellular injury (77).

## Conclusion

Organ perfusion has demonstrated it can serve as a viability assessment tool with current evidence suggesting normothermic perfusion is better suited. Indeed, although good quality evidence shows that injury markers and perfusion parameters during hypothermic kidney perfusion predict graft outcome, these markers lack the predictive accuracy needed in clinical practice. Little is known about the association of liver perfusate injury markers and perfusion parameters during hypothermic perfusion and this deserves further investigation. The recent large clinical trials, where livers were transplanted regardless of perfusate markers, provide valuable cohorts.

Normothermic perfusion, with a metabolically fully active organ, has been shown to be able to select viable grafts from those that were thought unsuitable for transplantation. Nevertheless, to date, there are no clear, validated and accurate markers to allow routine implementation of the technique in clinical settings. Data from larger studies are needed. Ideally, selection bias should be avoided by transplanting all organs that are perfused and blinding clinical teams to the viability assessment findings. However, as these studies would involve organs of doubtful viability, and therefore a reasonable chance of post-transplant failure, this obviously poses ethical concerns exposing patients to an increased risk of complications. One way would be to accumulate cases in large international registries so that a high enough number of cases with an undesirable outcome can be analysed together.

## Abbreviations

DCD, donation after circulatory death; DBD, donation after brain death; DGF, delayed graft function; PNF, primary non function; GST, glutathione S-transferase; H-FABP, heart-type fatty acid binding protein; NGAL, neutrophil gelatinase-associated lipocalin; FMN, flavin mononucleotide.

## References

[1] Israni AK Zaun D Rosendale JD Schaffhausen C McKinney W Snyder JJ. OPTN/SRTR 2019 Annual Data Report: Deceased Organ Donors. Am J Transpl (2021) 21 Suppl 2(Suppl. 2):521–58. 10.1111/ajt.16491 33595189

[2] Jochmans I van Rosmalen M Pirenne J Samuel U. Adult Liver Allocation in Eurotransplant. Transplantation (2017) 101(7):1542–50. 10.1097/tp.0000000000001631 28060242

[3] Heylen L Jochmans I Samuel U Tieken I Naesens M Pirenne J The Duration of Asystolic Ischemia Determines the Risk of Graft Failure after Circulatory-Dead Donor Kidney Transplantation: A Eurotransplant Cohort Study. Am J Transpl (2018) 18(4):881–9. 10.1111/ajt.14526 28980391

[4] Summers DM Watson CJE Pettigrew GJ Johnson RJ Collett D Neuberger JM Kidney Donation after Circulatory Death (DCD): State of the Art. Kidney Int (2015) 88(2):241–9. 10.1038/ki.2015.88 25786101

[5] Siedlecki A Irish W Brennan DC. Delayed Graft Function in the Kidney Transplant. Am J Transpl (2011) 11(11):2279–96. 10.1111/j.1600-6143.2011.03754.x PMC3280444 21929642

[6] Schröppel B Legendre C. Delayed Kidney Graft Function: from Mechanism to Translation. Kidney Int (2014) 86(2):251–8. 10.1038/ki.2014.18 24522494

[7] Jay CL Lyuksemburg V Ladner DP Wang E Caicedo JC Holl JL Ischemic Cholangiopathy after Controlled Donation after Cardiac Death Liver Transplantation. Ann Surg (2011) 253(2):259–64. 10.1097/sla.0b013e318204e658 21245668

[8] Ziogas IA Kakos CD Esagian SM Skarentzos K Alexopoulos SP Shingina A Liver Transplant after Donation from Controlled Circulatory Death versus Brain Death: A UNOS Database Analysis and Publication Bias Adjusted Meta-Analysis. Clin Transpl (2021) e14521. 10.1111/ctr.14521 34689372

[9] Jochmans I Akhtar MZ Nasralla D Kocabayoglu P Boffa C Kaisar M Past, Present, and Future of Dynamic Kidney and Liver Preservation and Resuscitation. Am J Transpl (2016) 16(9):2545–55. 10.1111/ajt.13778 26946212

[10] Belzer F Ashby BS Dunphy JE. 24-hour and 72-hour Preservation of Canine Kidneys. The Lancet (1967) 290(7515):536–9. 10.1016/s0140-6736(67)90498-9 4166894

[11] Belzer FO Ashby BS Gulyassy PF Powell M. Successful Seventeen-Hour Preservation and Transplantation of Human-Cadaver Kidney. N Engl J Med (1968) 278(11):608–10. 10.1056/nejm196803142781108 4866541

[12] McAnulty JF Ploeg RJ Southard JH Belzer FO. Successful Five-Day Perfusion Preservation of the Canine Kidney. Transplantation (1989) 47(1):37–41. 10.1097/00007890-198901000-00009 2643229

[13] Tingle SJ Figueiredo RS Moir JA Goodfellow M Talbot D Wilson CH. Machine Perfusion Preservation versus Static Cold Storage for Deceased Donor Kidney Transplantation. Cochrane Database Syst Rev (2019) 3:CD011671. 10.1002/14651858.CD011671.pub2 30875082 PMC6419919

[14] Hosgood SA Brown RJ Nicholson ML. Advances in Kidney Preservation Techniques and Their Application in Clinical Practice. Transplantation (2021) 105(11):e202–e214. 10.1097/tp.0000000000003679 33982904 PMC8549459

[15] Longchamp A Klauser A Songeon J Agius T Nastasi A Ruttiman R *Ex Vivo* Analysis of Kidney Graft Viability Using 31P Magnetic Resonance Imaging Spectroscopy. Transplantation (2020) 104(9):1825–31. 10.1097/tp.0000000000003323 32675744

[16] Nieuwenhuijs-Moeke GJ Pischke SE Berger SP Sanders JSF Pol RA Struys MMRF Ischemia and Reperfusion Injury in Kidney Transplantation: Relevant Mechanisms in Injury and Repair. J Clin Med (2020) 9(1). 10.3390/jcm9010253 PMC7019324 31963521

[17] Eltzschig HK Eckle T. Ischemia and Reperfusion-From Mechanism to Translation. Nat Med (2011) 17(11):1391–401. 10.1038/nm.2507 22064429 PMC3886192

[18] Mills EL Kelly B O'Neill LAJ. Mitochondria Are the Powerhouses of Immunity. Nat Immunol (2017) 18(5):488–98. 10.1038/ni.3704 28418387

[19] Haas M Loupy A Lefaucheur C Roufosse C Glotz D Seron D The Banff 2017 Kidney Meeting Report: Revised Diagnostic Criteria for Chronic Active T Cell-Mediated Rejection, Antibody‐mediated Rejection, and Prospects for Integrative Endpoints for Next‐generation Clinical Trials. Am J Transpl (2018) 18(2):293–307. 10.1111/ajt.14625 PMC5817248 29243394

[20] Wu WK Famure O Li Y Kim SJ. Delayed Graft Function and the Risk of Acute Rejection in the Modern Era of Kidney Transplantation. Kidney Int (2015) 88(4):851–8. 10.1038/ki.2015.190 26108067

[21] Lefaucheur C Gosset C Rabant M Viglietti D Verine J Aubert O T Cell-Mediated Rejection Is a Major Determinant of Inflammation in Scarred Areas in Kidney Allografts. Am J Transpl (2018) 18(2):377–90. 10.1111/ajt.14565 29086461

[22] Carcy R Cougnon M Poet M Durandy M Sicard A Counillon L Targeting Oxidative Stress, a Crucial challenge in Renal Transplantation Outcome. Free Radic Biol Med (2021) 169:258–70. 10.1016/j.freeradbiomed.2021.04.023 33892115

[23] Tejchman K Kotfis K Sieńko J. Biomarkers and Mechanisms of Oxidative Stress-Last 20 Years of Research with an Emphasis on Kidney Damage and Renal Transplantation. Int J Mol Sci (2021) 22(15). 10.3390/ijms22158010 PMC8347360 34360776

[24] Bellini MI Tortorici F Amabile MI D'Andrea V. Assessing Kidney Graft Viability and its Cells Metabolism during Machine Perfusion. Int J Mol Sci (2021) 22(3). 10.3390/ijms22031121 PMC7865666 33498732

[25] Jochmans I Brat A Davies L Hofker HS van de Leemkolk FEM Leuvenink HGD Oxygenated versus Standard Cold Perfusion Preservation in Kidney Transplantation (COMPARE): a Randomised, Double-Blind, Paired, Phase 3 Trial. The Lancet (2020) 396(10263):1653–62. 10.1016/s0140-6736(20)32411-9 33220737

[26] Guzzi F Knight SR Ploeg RJ Hunter JP. A Systematic Review to Identify whether Perfusate Biomarkers Produced during Hypothermic Machine Perfusion Can Predict Graft Outcomes in Kidney Transplantation. Transpl Int (2020) 33(6):590–602. 10.1111/tri.13593 32031281

[27] Moers C Varnav OC van Heurn E Jochmans I Kirste GR Rahmel A The Value of Machine Perfusion Perfusate Biomarkers for Predicting Kidney Transplant Outcome. Transplantation (2010) 90(9):966–73. 10.1097/tp.0b013e3181f5c40c 20861807

[28] Nagelschmidt M Minor T Gallinat A Moers C Jochmans I Pirenne J Lipid Peroxidation Products in Machine Perfusion of Older Donor Kidneys. J Surg Res (2013) 180(2):337–42. 10.1016/j.jss.2012.04.071 22626559

[29] Hall IE Bhangoo RS Reese PP Doshi MD Weng FL Hong K Glutathione S-Transferase Iso-Enzymes in Perfusate from Pumped Kidneys Are Associated with Delayed Graft Function. Am J Transpl (2014) 14(4):886–96. 10.1111/ajt.12635 PMC4051136 24612768

[30] Parikh CR Hall IE Bhangoo RS Ficek J Abt PL Thiessen-Philbrook H Associations of Perfusate Biomarkers and Pump Parameters with Delayed Graft Function and Deceased Donor Kidney Allograft Function. Am J Transpl (2016) 16(5):1526–39. 10.1111/ajt.13655 PMC4844819 26695524

[31] Hoogland ERP de Vries EE Christiaans MHL Winkens B Snoeijs MGJ van Heurn LWE. The Value of Machine Perfusion Biomarker Concentration in DCD Kidney Transplantations. Transplantation (2013) 95(4):603–10. 10.1097/tp.0b013e31827908e6 23296150

[32] Jochmans I Lerut E van Pelt J Monbaliu D Pirenne J. Circulating AST, H-FABP, and NGAL Are Early and Accurate Biomarkers of Graft Injury and Dysfunction in a Preclinical Model of Kidney Transplantation. Ann Surg (2011) 254(5):784–92. 10.1097/sla.0b013e3182368fa7 21997818

[33] Moser MAJ Arcand S Lin H-B Wojnarowicz C Sawicka J Banerjee T Protection of the Transplant Kidney from Preservation Injury by Inhibition of Matrix Metalloproteinases. PLoS One (2016) 11(6):e0157508. 10.1371/journal.pone.0157508 27327879 PMC4915675

[34] Gómez-Dos-Santos V Ramos-Muñoz E García-Bermejo ML Ruiz-Hernández M Rodríguez-Serrano EM Saiz-González A MicroRNAs in Kidney Machine Perfusion Fluid as Novel Biomarkers for Graft Function. Normalization Methods for miRNAs Profile Analysis. Transplant Proc (2019) 51(2):307–10. 10.1016/j.transproceed.2018.09.019 30879529

[35] de Vries EE Hoogland ERP Winkens B Snoeijs MG van Heurn LWE. Renovascular Resistance of Machine‐Perfused DCD Kidneys Is Associated with Primary Nonfunction. Am J Transplant (2011) 11(12):2685–91. 10.1111/j.1600-6143.2011.03755.x 21967629

[36] Mozes MF Skolek RB Korf BC. Use of Perfusion Parameters in Predicting Outcomes of Machine-Preserved Kidneys. Transplant Proc (2005) 37(1):350–1. 10.1016/j.transproceed.2005.01.058 15808640

[37] Guarrera JV Goldstein MJ Samstein B Henry S Reverte C Arrington B 'When Good Kidneys Pump Badly': Outcomes of Deceased Donor Renal Allografts with Poor Pulsatile Perfusion Characteristics. Transpl Int (2010) 23(4):444–6. 10.1111/j.1432-2277.2009.00970.x 19778343

[38] Sung RS Christensen LL Leichtman AB Greenstein SM Distant DA Wynn JJ Determinants of Discard of Expanded Criteria Donor Kidneys: Impact of Biopsy and Machine Perfusion. Am J Transpl (2008) 8(4):783–92. 10.1111/j.1600-6143.2008.02157.x 18294347

[39] Jochmans I Moers C Smits JM Leuvenink HGD Treckmann J Paul A The Prognostic Value of Renal Resistance during Hypothermic Machine Perfusion of Deceased Donor Kidneys. Am J Transpl (2011) 11(10):2214–20. 10.1111/j.1600-6143.2011.03685.x 21834917

[40] Jochmans I Nicholson ML Hosgood SA. Kidney Perfusion. Curr Opin Organ Transpl (2017) 22(3):260–6. 10.1097/mot.0000000000000405 28301386

[41] Hosgood SA Saeb-Parsy K Wilson C Callaghan C Collett D Nicholson ML. Protocol of a Randomised Controlled, Open-Label Trial of *Ex Vivo* Normothermic Perfusion versus Static Cold Storage in Donation after Circulatory Death Renal Transplantation. BMJ open (2017) 7(1):e012237. 10.1136/bmjopen-2016-012237 PMC5278243 28115329

[42] Kaths JM Echeverri J Goldaracena N Louis KS Chun Y-M Linares I Eight-Hour Continuous Normothermic *Ex Vivo* Kidney Perfusion Is a Safe Preservation Technique for Kidney Transplantation. Transplantation (2016) 100(9):1862–70. 10.1097/tp.0000000000001299 27479157

[43] Urbanellis P Hamar M Kaths JM Kollmann D Linares I Mazilescu L Normothermic *Ex Vivo* Kidney Perfusion Improves Early DCD Graft Function Compared with Hypothermic Machine Perfusion and Static Cold Storage. Transplantation (2020) 104(5):947–55. 10.1097/tp.0000000000003066 31815900

[44] Hosgood SA Nicholson ML. First in Man Renal Transplantation after *Ex Vivo* Normothermic Perfusion. Transplantation (2011) 92(7):735–8. 10.1097/tp.0b013e31822d4e04 21841540

[45] Hosgood SA Nicholson ML. Ex VivoNormothermic Perfusion of Declined Human Kidneys after InadequateIn SituPerfusion. Am J Transpl (2014) 14(2):490–1. 10.1111/ajt.12568 24330455

[46] Hosgood SA Thompson E Moore T Wilson CH Nicholson ML. Normothermic Machine Perfusion for the Assessment and Transplantation of Declined Human Kidneys from Donation after Circulatory Death Donors. Br J Surg (2018) 105(4):388–94. 10.1002/bjs.10733 29210064 PMC5887977

[47] Patel M Hosgood S Nicholson ML. The Effects of Arterial Pressure during Normothermic Kidney Perfusion. J Surg Res (2014) 191(2):463–8. 10.1016/j.jss.2014.04.003 24811916

[48] De Beule J Jochmans I. Kidney Perfusion as an Organ Quality Assessment Tool-Are We Counting Our Chickens before They Have Hatched. Jcm (2020) 9(3):879. 10.3390/jcm9030879 PMC7141526 32210197

[49] Hosgood SA Nicholson ML. An Assessment of Urinary Biomarkers in a Series of Declined Human Kidneys Measured during *Ex Vivo* Normothermic Kidney Perfusion. Transplantation (2017) 101(9):2120–5. 10.1097/tp.0000000000001504 27681269

[50] Schutter R Lantinga VA Hamelink TL Pool MBF Varsseveld OC Potze JH Magnetic Resonance Imaging Assessment of Renal Flow Distribution Patterns during *Ex Vivo* Normothermic Machine Perfusion in Porcine and Human Kidneys. Transpl Int (2021) 34(9):1643–55. 10.1111/tri.13991 34448269 PMC9290094

[51] Hosgood SA Nicholson ML. A Short Period of Normothermic Machine Perfusion May Not Be Able to Predict Primary Nonfunction in Uncontrolled Circulatory Death Kidneys. Transplantation (2021) 105(1):e11–e12. 10.1097/tp.0000000000003415 33350632

[52] Ikeda T Yanaga K Lebeau G Higashi H Kakizoe S Starzl TE. Hemodynamic and Biochemical Changes during Normothermic and Hypothermic Sanguinous Perfusion of the Porcine Hepatic Graft. Transplantation (1990) 50(4):564–7. 10.1097/00007890-199010000-00006 2219274 PMC2967246

[53] Yanaga K Makowka L Lebeau G Hwang RR Shimada M Kakizoe S A New Liver Perfusion and Preservation System for Transplantation Research in Large Animals. J Invest Surg (1990) 3(1):65–75. 10.3109/08941939009140337 2282350 PMC2956500

[54] Brettschneider Cgg L Starzl TE. Experimental and Clinical Preservation of Orthotopic Liver Homografts. In: Norman J, editor. Organ Perfusion and Preservation. New York: Appleton-Century-Crofts (1968). p. 271–84.

[55] Olthoff KM Kulik L Samstein B Kaminski M Abecassis M Emond J Validation of a Current Definition of Early Allograft Dysfunction in Liver Transplant Recipients and Analysis of Risk Factors. Liver Transpl (2010) 16(8):943–9. 10.1002/lt.22091 20677285

[56] Jochmans I Fieuws S Monbaliu D Pirenne J. "Model for Early Allograft Function" Outperforms "Early Allograft Dysfunction" as a Predictor of Transplant Survival. Transplantation (2017) 101(8):e258–e264. 10.1097/tp.0000000000001833 28557956

[57] Pareja E Cortes M Hervás D Mir J Valdivieso A Castell JV A Score Model for the Continuous Grading of Early Allograft Dysfunction Severity. Liver Transpl (2015) 21(1):38–46. 10.1002/lt.23990 25204890

[58] Oh CK Sawyer RG Pelletier SJ Pruett TL Sanfey HA. Independent Predictors for Primary Non-function after Liver Transplantation. Yonsei Med J (2004) 45(6):1155–61. 10.3349/ymj.2004.45.6.1155 15627312

[59] op den Dries S Westerkamp AC Karimian N Gouw ASH Bruinsma BG Markmann JF Injury to Peribiliary Glands and Vascular Plexus before Liver Transplantation Predicts Formation of Non-anastomotic Biliary Strictures. J Hepatol (2014) 60(6):1172–9. 10.1016/j.jhep.2014.02.010 24560661

[60] Mourad MM Algarni A Liossis C Bramhall SR. Aetiology and Risk Factors of Ischaemic Cholangiopathy after Liver Transplantation. Wjg (2014) 20(20):6159–69. 10.3748/wjg.v20.i20.6159 24876737 PMC4033454

[61] Schlegel A Rougemont Od. Graf R Clavien P-A Dutkowski P. Protective Mechanisms of End-Ischemic Cold Machine Perfusion in DCD Liver Grafts. J Hepatol (2013) 58(2):278–86. 10.1016/j.jhep.2012.10.004 23063573

[62] Patrono D Roggio D Mazzeo AT Catalano G Mazza E Rizza G Clinical Assessment of Liver Metabolism during Hypothermic Oxygenated Machine Perfusion Using Microdialysis. JArtif Organs (2022) 46(2):281–95. 10.1111/aor.14066 PMC9292750 34516020

[63] Guarrera JV Henry SD Samstein B Odeh-Ramadan R Kinkhabwala M Goldstein MJ Hypothermic Machine Preservation in Human Liver Transplantation: the First Clinical Series. Am J Transpl (2010) 10(2):372–81. 10.1111/j.1600-6143.2009.02932.x 19958323

[64] van Rijn R Karimian N Matton APM Burlage LC Westerkamp AC van den Berg AP Dual Hypothermic Oxygenated Machine Perfusion in Liver Transplants Donated after Circulatory Death. Br J Surg (2017) 104(7):907–17. 10.1002/bjs.10515 28394402 PMC5484999

[65] van Rijn R Schurink IJ de Vries Y van den Berg AP Cortes Cerisuelo M Darwish Murad S Hypothermic Machine Perfusion in Liver Transplantation - A Randomized Trial. N Engl J Med (2021) 384(15):1391–401. 10.1056/nejmoa2031532 33626248

[66] Czigany Z Pratschke J Froněk J Guba M Schöning W Raptis DA Hypothermic Oxygenated Machine Perfusion Reduces Early Allograft Injury and Improves Post-transplant Outcomes in Extended Criteria Donation Liver Transplantation from Donation after Brain Death: Results from a Multicenter Randomized Controlled Trial (HOPE ECD-DBD). Ann Surg (2021) 274(5):705–12. 10.1097/SLA.0000000000005110 34334635

[67] Guarrera JV Henry SD Samstein B Reznik E Musat C Lukose TI Hypothermic Machine Preservation Facilitates Successful Transplantation of "orphan" Extended Criteria Donor Livers. Am J Transpl (2015) 15(1):161–9. 10.1111/ajt.12958 25521639

[68] Patrono D Catalano G Rizza G Lavorato N Berchialla P Gambella A Perfusate Analysis during Dual Hypothermic Oxygenated Machine Perfusion of Liver Grafts: Correlations with Donor Factors and Early Outcomes. Transplantation (2020) 104(9):1929–42. 10.1097/tp.0000000000003398 32769628

[69] Muller X Schlegel A Kron P Eshmuminov D Würdinger M Meierhofer D Novel Real-Time Prediction of Liver Graft Function during Hypothermic Oxygenated Machine Perfusion before Liver Transplantation. Ann Surg (2019) 270(5):783–90. 10.1097/sla.0000000000003513 31592808

[70] Patrono D Surra A Catalano G Rizza G Berchialla P Martini S Hypothermic Oxygenated Machine Perfusion of Liver Grafts from Brain-Dead Donors. Sci Rep (2019) 9(1):9337. 10.1038/s41598-019-45843-3 31249370 PMC6597580

[71] Nasralla D Coussios CC Coussios CC Mergental H Akhtar MZ Butler AJ A Randomized Trial of Normothermic Preservation in Liver Transplantation. Nature (2018) 557(7703):50–6. 10.1038/s41586-018-0047-9 29670285

[72] Martins PN Rizzari MD Ghinolfi D Jochmans I Attia M Jalan R Design, Analysis, and Pitfalls of Clinical Trials Using *Ex Situ* Liver Machine Perfusion: The International Liver Transplantation Society Consensus Guidelines. Transplantation (2021) 105(4):796–815. 10.1097/tp.0000000000003573 33760791

[73] Mergental H Perera MTPR Laing RW Muiesan P Isaac JR Smith A Transplantation of Declined Liver Allografts Following Normothermic *Ex-Situ* Evaluation. Am J Transpl (2016) 16(11):3235–45. 10.1111/ajt.13875 27192971

[74] Ravikumar R Jassem W Mergental H Heaton N Mirza D Perera MTPR Liver Transplantation AfterEx VivoNormothermic Machine Preservation: A Phase 1 (First-In-Man) Clinical Trial. Am J Transpl (2016) 16(6):1779–87. 10.1111/ajt.13708 26752191

[75] Bral M Gala-Lopez B Bigam D Kneteman N Malcolm A Livingstone S Preliminary Single-Center Canadian Experience of Human NormothermicEx VivoLiver Perfusion: Results of a Clinical Trial. Am J Transpl (2017) 17(4):1071–80. 10.1111/ajt.14049 27639262

[76] Watson CJE Kosmoliaptsis V Randle LV Gimson AE Brais R Klinck JR Normothermic Perfusion in the Assessment and Preservation of Declined Livers before Transplantation. Transplantation (2017) 101(5):1084–98. 10.1097/tp.0000000000001661 28437389 PMC5642347

[77] Watson CJE Kosmoliaptsis V Pley C Randle L Fear C Crick K Observations on the *Ex Situ* Perfusion of Livers for Transplantation. Am J Transpl (2018) 18(8):2005–20. 10.1111/ajt.14687 PMC6099221 29419931

[78] Matton APM de Vries Y Burlage LC van Rijn R Fujiyoshi M de Meijer VE Biliary Bicarbonate, pH, and Glucose Are Suitable Biomarkers of Biliary Viability during *Ex Situ* Normothermic Machine Perfusion of Human Donor Livers. Transplantation (2019) 103(7):1405–13. 10.1097/tp.0000000000002500 30395120 PMC6613725

[79] van Leeuwen OB de Vries Y Fujiyoshi M Nijsten MWN Ubbink R Pelgrim GJ Transplantation of High-Risk Donor Livers after *Ex Situ* Resuscitation and Assessment Using Combined Hypo- and Normothermic Machine Perfusion. Ann Surg (2019) 270(5):906–14. 10.1097/sla.0000000000003540 31633615

[80] Mergental H Laing RW Kirkham AJ Perera MTPR Boteon YL Attard J Transplantation of Discarded Livers Following Viability Testing with Normothermic Machine Perfusion. Nat Commun (2020) 11(1):2939. 10.1038/s41467-020-16251-3 32546694 PMC7298000

[81] Selzner M Goldaracena N Echeverri J Kaths JM Linares I Selzner N Normothermic *Ex Vivo* Liver Perfusion Using Steen Solution as Perfusate for Human Liver Transplantation: First North American Results. Liver Transpl (2016) 22(11):1501–8. 10.1002/lt.24499 27339754

[82] Liu Q Nassar A Farias K Buccini L Baldwin W Mangino M Sanguineous Normothermic Machine Perfusion Improves Hemodynamics and Biliary Epithelial Regeneration in Donation after Cardiac Death Porcine Livers. Liver Transpl (2014) 20(8):987–99. 10.1002/lt.23906 24805852 PMC4117809

[83] Patrono D Cussa D Rigo F Romagnoli R. Liver Machine Perfusion Survey G. Heterogeneous Indications and the Need for Viability Assessment: An International Survey on the Use of Machine Perfusion in Liver Transplantation. Artif Organs (2022). 46(2):296–305. 10.1111/aor.14061 34460943 PMC9291461

[84] Watson CJE Jochmans I. From "Gut Feeling" to Objectivity: Machine Preservation of the Liver as a Tool to Assess Organ Viability. Curr Transpl Rep (2018) 5(1):72–81. 10.1007/s40472-018-0178-9 29564205 PMC5843692

[85] op den Dries S Karimian N Sutton ME Westerkamp AC Nijsten MWN Gouw ASH Ex vivoNormothermic Machine Perfusion and Viability Testing of Discarded Human Donor Livers. Am J Transpl (2013) 13(5):1327–35. 10.1111/ajt.12187 23463950

[86] Fodor M Woerdehoff A Peter W Esser H Oberhuber R Margreiter C Reassessment of Relevance and Predictive Value of Parameters Indicating Early Graft Dysfunction in Liver Transplantation: AST Is a Weak, but Bilirubin and INR Strong Predictors of Mortality. Front Surg (2021) 8:693288. 10.3389/fsurg.2021.693288 34869549 PMC8634944

[87] Martins PN Buchwald JE Mergental H Vargas L Quintini C. The Role of Normothermic Machine Perfusion in Liver Transplantation. Int J Surg (2020) 82:52–60. 10.1016/j.ijsu.2020.05.026 32417462

[88] Brosnan ME Brosnan JT. Hepatic Glutamate Metabolism: a Tale of 2 Hepatocytes. Am J Clin Nutr (2009) 90(3):857S–861S. 10.3945/ajcn.2009.27462z 19625684

[89] Gilbo N Jacquemin M Nasralla D Lazzaro S Libbrecht L Lavend'homme R Coagulation Factors Accumulate during Normothermic Liver Machine Perfusion Regardless of Donor Type and Severity of Ischemic Injury. Transplantation (2021) (Online ahead of print). 10.1097/TP.0000000000003763 33756546

[90] Karangwa SA Adelmeijer J Matton APM de Meijer VE Lisman T Porte RJ. Production of Physiologically Relevant Quantities of Hemostatic Proteins during *Ex Situ* Normothermic Machine Perfusion of Human Livers. Liver Transpl (2018) 24(9):1298–302. 10.1002/lt.25290 30125455

[91] Sutton ME op den Dries S Karimian N Weeder PD de Boer MT Wiersema-Buist J Criteria for Viability Assessment of Discarded Human Donor Livers during *Ex Vivo* Normothermic Machine Perfusion. PLoS One (2014) 9(11):e110642. 10.1371/journal.pone.0110642 25369327 PMC4219693

[92] Brüggenwirth IMA Porte RJ Martins PN. Bile Composition as a Diagnostic and Prognostic Tool in Liver Transplantation. Liver Transpl (2020) 26(9):1177–87. 10.1002/lt.25771 32246581

[93] Concepcion AR Lopez M Ardura-Fabregat A Medina JF. Role of AE2 for pHi Regulation in Biliary Epithelial Cells. Front Physiol (2013) 4:413. 10.3389/fphys.2013.00413 24478713 PMC3894451

[94] Gaurav R Atulugama N Swift L Butler AJ Upponi S Brais R Bile Biochemistry Following Liver Reperfusion in the Recipient and its Association with Cholangiopathy. Liver Transpl (2020) 26(8):1000–9. 10.1002/lt.25738 32108995 PMC7497270

[95] Hosgood SA Barlow AD Dormer J Nicholson ML. The Use of *Ex-Vivo* Normothermic Perfusion for the Resuscitation and Assessment of Human Kidneys Discarded Because of Inadequate *In Situ* Perfusion. J Transl Med (2015) 13:329. 10.1186/s12967-015-0691-x 26474973 PMC4609141

